# Solutions for the quasi-linear elliptic problems involving the critical Sobolev exponent

**DOI:** 10.1186/s13660-017-1492-y

**Published:** 2017-09-12

**Authors:** Yanbin Sang, Siman Guo

**Affiliations:** Department of Mathematics, School of Science, North University of China, Taiyuan, Shanxi 030051 China

**Keywords:** quasi-linear elliptic problems, Nehari manifold, positive solution, best Sobolev constant

## Abstract

In this article, we study the existence and multiplicity of positive solutions for the quasi-linear elliptic problems involving critical Sobolev exponent and a Hardy term. The main tools adopted in our proofs are the concentration compactness principle and Nehari manifold.

## Introduction

In this article, we consider the following quasi-linear elliptic problem:

1.1 $$ \textstyle\begin{cases} -\Delta_{p}u-\mu\frac{ \vert u \vert ^{p-2}u}{ \vert x \vert ^{p}}= \vert u \vert ^{p^{*}-2}u+\beta \vert x \vert ^{\alpha-p} \vert u \vert ^{p-2}u+\lambda \vert u \vert ^{q-2}u\quad \mbox{in }\Omega, \\ u=0\quad \mbox{on }\partial\Omega, \end{cases} $$

where $\Omega\subset\mathbb{R}^{N}\ (N\geq3)$ is a bounded domain with the smooth boundary *∂*Ω such that $0\in\Omega$. $\Delta _{p}u=\operatorname{div}( \vert \nabla u \vert ^{p-2}\nabla u)$ is the *p*-Laplacian operator of *u*, $1< p< N, \lambda>0$ is a positive real number. $0\leq\mu<\overline {\mu}$ ($\overline{\mu}=\frac{(N-p)^{p}}{p}$ is the best Hardy constant). $1< q< p$ and $p^{*}=\frac{Np}{N-p}$ is the critical Sobolev exponent. $0<\alpha<p-1$, $0<\beta<\beta_{1}$ ($\beta_{1}$ is the first eigenvalue that $-\Delta_{p}u-\mu\frac{ \vert u \vert ^{p-2}u}{ \vert x \vert ^{p}}= \vert x \vert ^{\alpha -p} \vert u \vert ^{p-2}u$ under Dirichlet boundary condition).

### Definition 1.1

The function $u\in W_{0}^{1,p}(\Omega)$ is called a weak solution of (1.1) if *u* satisfies

1.2 $$\begin{aligned} &\int_{\Omega}\biggl( \vert \nabla u \vert ^{p-2}\nabla u\cdot\nabla v-\mu\frac{ \vert u \vert ^{p-2}uv}{ \vert x \vert ^{p}}\biggr)\,dx \\ &\quad = \int_{\Omega}\bigl( \vert u \vert ^{p^{*}-2}uv+\beta \vert x \vert ^{\alpha-p} \vert u \vert ^{p-2}uv+\lambda \vert u \vert ^{q-2}uv\bigr)\,dx \end{aligned}$$

for all $v\in W_{0}^{1,p}(\Omega)$.

In this paper, we use the following norm of $W_{0}^{1,p}(\Omega)$:

$$\Vert u \Vert =\biggl( \int_{\Omega}\biggl( \vert \nabla u \vert ^{p}-\mu \frac{ \vert u \vert ^{p}}{ \vert x \vert ^{p}}\biggr)\,dx\biggr)^{\frac{1}{p}}. $$

By the Hardy inequality (see [1, 2])

$$\int_{\Omega}\frac{ \vert u \vert ^{p}}{ \vert x \vert ^{p}}\,dx\leq\frac{1}{\overline{\mu}} \int _{\Omega} \vert \nabla u \vert ^{p}\,dx, \quad\forall u\in W_{0}^{1,p}(\Omega), $$

so this norm is equivalent to $(\int_{\Omega} \vert \nabla u \vert ^{p}\,dx)^{\frac {1}{p}}$, the usual norm in $W_{0}^{1,p}(\Omega)$.

The norm in $L^{p}(\Omega)$ is represented by $\Vert u \Vert _{p}=(\int_{\Omega } \vert u \vert ^{p}\,dx)^{\frac{1}{p}}$. According to Hardy inequality, the following best Sobolev constant is well defined for $1< p< N$, and $0\leq\mu<\overline{\mu}$:

1.3 $$ S_{\mu,0}=\inf_{u\in W_{0}^{1,p}(\Omega)\backslash\{0\} }\frac{\int_{\Omega}( \vert \nabla u \vert ^{p}-\mu\frac{ \vert u \vert ^{p}}{ \vert x \vert ^{p}})\,dx}{(\int _{\Omega} \vert u \vert ^{p^{*}}\,dx)^{\frac{p}{p^{*}}}}. $$

The quasi-linear problems on Hardy inequality have been studied extensively, either in the smooth bounded domain or in the whole space $\mathbb{R}^{N}$. More and more excellent results have been obtained, which provide us opportunities to understand the singular problems. However, compared with the semilinear case, the quasi-linear problems related to Hardy inequality are more complicated [3–16]. Abdellaoui, Felli and Peral [3] considered the extremal function which achieves the best constant $S_{\mu,0}$, and gave the properties of the extremal functions. The conclusions obtained in [3] can be applied in the problems with critical Sobolev exponent and Hardy term.

Wang, Wei and Kang [10] investigated the following problem:

1.4 $$ \textstyle\begin{cases} -\Delta_{p} u-\lambda\frac{ \vert u \vert ^{p-2}}{ \vert x \vert ^{p}}u=\mu f(x) \vert u \vert ^{q-2} u+g(x) \vert u \vert ^{p^{*}-2} u,\quad x\in\Omega,\\ u(x)=0,\quad x\in\partial\Omega, \end{cases} $$

where $1< q< p, \mu>0$, *f* and *g* are non-negative functions and $p^{*}=\frac{Np}{N-p}$ is the critical Sobolev exponent. The property of the Nehari manifold was used to prove the existence of multiple positive solutions for (1.4). Furthermore, Hsu [11, 12] improved and complemented the main results obtained in [10]. Recently, Goyal and Sreenadh [13] investigated a class of singular *N*-Laplacian problems with exponential nonlinearities in $\mathbb{R}^{N}$. Very recently, Xiang [14] established the asymptotic estimates of weak solutions for *p*-Laplacian equation with Hardy term and critical Sobolev exponent.

We should mention that Liu, Guo and Lei [17] studied the existence and multiplicity of positive solutions of Kirchhoff equation with critical exponential nonlinearity. Inspired by [17, 18], we study the problem (1.1) on critical Sobolev exponent. Comparing with the main results obtained in [4, 6, 10–12], in this paper, on the one hand, we will analysis the effect of $\beta \vert x \vert ^{\alpha-p} \vert u \vert ^{p-2}u$, and the more careful estimates are needed. On the other hand, we establish an lower bound for $\lambda_{*}$ ($\lambda_{*}$ is defined in Theorem 1.1).

Define the energy functional associated to problem (1.1) as follows:

1.5 $$ I_{\lambda}(u)=\frac{1}{p} \Vert u \Vert ^{p}-\frac{\beta}{p} \int_{\Omega } \vert u \vert ^{p} \vert x \vert ^{\alpha-p}\,dx- \frac{1}{p^{*}} \int_{\Omega} \vert u \vert ^{p^{*}}\,dx- \frac{\lambda}{q} \int_{\Omega } \vert u \vert ^{q}\,dx. $$

We obtain the following result.

### Theorem 1.1

*Suppose that* $1< q< p$, $0<\alpha <p-1$. *Then there exists* $\lambda_{*}>0$ *such that problem* (1.1) *admits at least two solutions and one of the solutions is a ground state solution for all* $\lambda\in(0,\lambda_{*})$.

## Preliminaries

Firstly, we introduce the Nehari manifold

$$\mathcal{N}_{\lambda}=\bigl\{ u\in W_{0}^{1,p}(\Omega) \backslash\{0\}:\bigl\langle I_{\lambda}^{\prime}(u),u\bigr\rangle =0 \bigr\} . $$

Furthermore $u\in\mathcal{N}_{\lambda}$ if and only if

2.1 $$ \Vert u \Vert ^{p}- \int_{\Omega} \vert u \vert ^{p^{*}}\,dx-\beta \int_{\Omega } \vert u \vert ^{p} \vert x \vert ^{\alpha-p}\,dx -\lambda \int_{\Omega} \vert u \vert ^{q}\,dx=0. $$

Let

$$\psi(u):= \Vert u \Vert ^{p}-\beta \int_{\Omega} \vert u \vert ^{p} \vert x \vert ^{\alpha-p}\,dx - \int_{\Omega} \vert u \vert ^{p^{*}}\,dx -\lambda \int_{\Omega} \vert u \vert ^{q}\,dx, $$

then

$$\bigl\langle \psi^{\prime}(u), u\bigr\rangle =p \Vert u \Vert ^{p}-p\beta \int_{\Omega } \vert u \vert ^{p} \vert x \vert ^{\alpha-p}\,dx -p^{*} \int_{\Omega} \vert u \vert ^{p^{*}}\,dx-q\lambda \int_{\Omega} \vert u \vert ^{q}\,dx. $$

$\mathcal{N}_{\lambda}$ can be divided into the following three parts:

2.2 $$\begin{aligned} & \mathcal{N}_{\lambda}^{+}=\biggl\{ u\in \mathcal{N}_{\lambda}:p \Vert u \Vert ^{p}-p\beta \int_{\Omega} \vert x \vert ^{\alpha-p} \vert u \vert ^{p}\,dx \\ &\phantom{\mathcal{N}_{\lambda}^{+}=}{}-p^{*} \int_{\Omega} \vert u \vert ^{p^{*}}\,dx-q\lambda \int_{\Omega} \vert u \vert ^{q}\,dx>0\biggr\} , \end{aligned}$$

2.3 $$\begin{aligned} &\mathcal{N}_{\lambda}^{0}=\biggl\{ u\in \mathcal{N}_{\lambda}:p \Vert u \Vert ^{p}-p\beta \int_{\Omega} \vert x \vert ^{\alpha-p} \vert u \vert ^{p}\,dx \\ &\phantom{\mathcal{N}_{\lambda}^{0}=}{}-p^{*} \int_{\Omega} \vert u \vert ^{p^{*}}\,dx-q\lambda \int_{\Omega} \vert u \vert ^{q}\,dx=0\biggr\} , \end{aligned}$$

2.4 $$\begin{aligned} &\mathcal{N}_{\lambda}^{-}=\biggl\{ u\in \mathcal{N}_{\lambda}:p \Vert u \Vert ^{p}-p\beta \int_{\Omega} \vert x \vert ^{\alpha-p} \vert u \vert ^{p}\,dx \\ &\phantom{\mathcal{N}_{\lambda}^{0}=}{} -p^{*} \int_{\Omega} \vert u \vert ^{p^{*}}\,dx-q\lambda \int_{\Omega} \vert u \vert ^{q}\,dx< 0\biggr\} . \end{aligned}$$

Applying the Hölder inequality and the Sobolev inequality, for all $u\in W_{0}^{1,p}(\Omega)\backslash\{0\}$ we have

2.5 $$ \int_{\Omega} \vert u \vert ^{q}\,dx\leq\biggl( \int_{\Omega} \vert u \vert ^{q\cdot\frac {p^{*}}{q}}\,dx \biggr)^{\frac{q}{p^{*}}}\biggl( \int_{\Omega}1\,dx\biggr)^{1-\frac{q}{p^{*}}} = \vert \Omega \vert ^{\frac{p^{*}-q}{p^{*}}}\biggl( \int_{\Omega} \vert u \vert ^{p^{*}}\,dx \biggr)^{\frac{q}{p^{*}}}. $$

### Lemma 2.1

*Assume that* $\lambda\in(0,T_{1})$ *with*

$$T_{1}=\frac{(\frac{(\beta_{1} -\beta)(p-p^{*})}{\beta_{1} (q-p^{*})})^{\frac {q-p^{*}}{p-p^{*}}}(\frac{q-p}{p-p^{*}})^{\frac{q-p}{p-p^{*}}}S_{\mu,0}^{\frac {q-p^{*}}{p-p^{*}}}}{ \vert \Omega \vert ^{\frac{p^{*} -q}{p^{*}}}}. $$

*Then* (i) $\mathcal{N}_{\lambda}^{\pm}\neq\emptyset$, *and* (ii) $\mathcal {N}_{\lambda}^{0}=\emptyset$.

### Proof

(i) We define a function $\Phi\in C(\mathbb {R}^{+},\mathbb{R})$ by

$$\Phi(s)=\biggl(1-\frac{\beta}{\beta_{1}}\biggr)s^{p-p^{*}} \Vert u \Vert ^{p}-\lambda s^{q-p^{*}} \int_{\Omega} \vert u \vert ^{q}\,dx. $$

Let $\Phi^{\prime}(s)=0$, that is,

$$\Phi^{\prime}(s)=\biggl(1-\frac{\beta}{\beta_{1}}\biggr) \bigl(p-p^{*}\bigr)s^{p-p^{*}-1} \Vert u \Vert ^{p}- \lambda\bigl(q-p^{*}\bigr)s^{q-p^{*}-1} \int_{\Omega} \vert u \vert ^{q}\,dx=0. $$

We can deduce that

$$s_{\max}:=s=\biggl[\frac{(\beta_{1} -\beta)(p-p^{*}) \Vert u \Vert ^{p}}{\beta_{1} \lambda (q-p^{*})\int_{\Omega} \vert u \vert ^{q}\,dx}\biggr] ^{\frac{1}{q-p}}. $$

It is easy to check that $\Phi^{\prime}(s)>0$ for all $0< s< s_{\max}$ and $\Phi^{\prime}(s)<0$ for all $s>s_{\max}$. Consequently, $\Phi(s)$ attains its maximum at $s_{\max}$, that is,

$$\begin{aligned} \Phi(s_{\max})={}&\biggl(1-\frac{\beta}{\beta_{1}}\biggr)\biggl\{ \biggl[ \frac {(\beta_{1} -\beta)(p-p^{*}) \Vert u \Vert ^{p}}{\beta_{1} \lambda(q-p^{*}) \int_{\Omega} \vert u \vert ^{q}\,dx}\biggr]^{\frac{1}{q-p}}\biggr\} ^{p-p^{*}} \Vert u \Vert ^{p} \\ &{}-\lambda\biggl\{ \biggl[\frac{(\beta_{1} -\beta)(p-p^{*}) \Vert u \Vert ^{p}}{ \beta_{1} \lambda(q-p^{*})\int_{\Omega} \vert u \vert ^{q}\,dx}\biggr]^{\frac{1}{q-p}}\biggr\} ^{q-p^{*}} \int_{\Omega} \vert u \vert ^{q}\,dx \\ ={}&\biggl(\frac{(\beta_{1} -\beta )(p-p^{*})}{\beta_{1}(q-p^{*})}\biggr)^{\frac{q-p^{*}}{q-p}}\biggl(\frac{q-p}{p-p^{*}}\biggr) \frac{ \Vert u \Vert ^{\frac{p(q-p^{*})}{q-p}}}{ (\lambda\int_{\Omega} \vert u \vert ^{q}\,dx)^{\frac{p-p^{*}}{q-p}}}. \end{aligned}$$

Since

$$\begin{aligned} \widetilde{\Phi}(s)&:=s^{p-p^{*}} \Vert u \Vert ^{p}-\beta s^{p-p^{*}} \int_{\Omega} \vert u \vert ^{p} \vert x \vert ^{\alpha-p}\,dx-\lambda s^{q-p^{*}} \int_{\Omega} \vert u \vert ^{q}\,dx \\ &\geq s^{p-p^{*}}\biggl(1-\frac{\beta}{\beta_{1}}\biggr) \Vert u \Vert ^{p}-\lambda s^{q-p^{*}} \int_{\Omega} \vert u \vert ^{q}\,dx. \end{aligned}$$

By (1.3) and (2.5), we have

$$\begin{aligned} &\widetilde{\Phi}(s_{\max}) - \int_{\Omega} \vert u \vert ^{p^{*}}\,dx \\ &\quad\geq\Phi(s_{\max}) - \int_{\Omega} \vert u \vert ^{p^{*}}\,dx \\ &\quad=\biggl(\frac{(\beta_{1} -\beta)(p-p^{*})}{\beta_{1} (q-p^{*})}\biggr)^{\frac{q-p^{*}}{q-p}} \biggl(\frac{q-p}{p-p^{*}} \biggr)\frac{ \Vert u \Vert ^{\frac{p(q-p^{*})}{q-p}}}{ (\lambda\int_{\Omega} \vert u \vert ^{q}\,dx) ^{\frac{p-p^{*}}{q-p}}}- \int_{\Omega} \vert u \vert ^{p^{*}}\,dx \\ &\quad>\biggl(\frac{(\beta_{1} -\beta)(p-p^{*})}{\beta_{1} (q-p^{*})}\biggr)^{\frac{q-p^{*}}{q-p}} \biggl(\frac{q-p}{p-p^{*}} \biggr)\frac{ \Vert u \Vert ^{\frac{p(q-p^{*})}{q-p}}}{ [\lambda \vert \Omega \vert ^{\frac{p^{*}-q}{p^{*}}}(\int_{\Omega } \vert u \vert ^{p^{*}}\,dx)^{\frac{q}{p^{*}}}]^{\frac{p-p^{*}}{q-p}} }- \int_{\Omega} \vert u \vert ^{p^{*}}\,dx \\ &\quad= \biggl\{ \biggl(\frac{(\beta_{1} -\beta)(p-p^{*})}{\beta_{1} (q-p^{*})}\biggr)^{\frac{q-p^{*}}{q-p}} \biggl( \frac{q-p}{p-p^{*}}\biggr)\frac{1}{ [\lambda \vert \Omega \vert ^{\frac{p^{*}-q}{p^{*}}}]^{\frac{p-p^{*}}{q-p}}} \biggl(\frac{ \Vert u \Vert ^{p}}{ (\int_{\Omega} \vert u \vert ^{p^{*}}\,dx)^{\frac{p}{p^{*}}}} \biggr)^{\frac {q-p^{*}}{q-p}}-1\biggr\} \\ &\qquad{}\times\int_{\Omega} \vert u \vert ^{p^{*}}\,dx \\ &\quad\geq \biggl\{ \biggl(\frac{(\beta_{1} -\beta)(p-p^{*})}{\beta_{1} (q-p^{*})}\biggr)^{\frac{q-p^{*}}{q-p}} \biggl( \frac{q-p}{p-p^{*}}\biggr)\frac{1}{ [\lambda \vert \Omega \vert ^{\frac{p^{*}-q}{p^{*}}}]^{\frac{p-p^{*}}{q-p}}} S_{\mu,0}^{\frac{q-p^{*}}{q-p}}-1 \biggr\} \int_{\Omega} \vert u \vert ^{p^{*}}\,dx \\ &\quad>0, \end{aligned}$$

where $0<\lambda<T_{1}$. Thus, there exist constants $s^{+}$ and $s^{-}$ such that

$$0< s^{+}=s^{+}(u)< s_{\max}< s^{-}=s^{-}(u),\quad s^{+}u\in\mathcal {N}_{\lambda}^{+} \mbox{ and } s^{-}u\in\mathcal{N}_{\lambda}^{-}. $$

(ii) We prove that $\mathcal{N}_{\lambda}^{0}=\emptyset$ for all $\lambda\in(0,T_{1})$. By contradiction, assume that there exists $u_{0}\neq 0$ such that $u_{0}\in\mathcal{N}_{\lambda}^{0}$. From (2.1), we have

2.6 $$ \Vert u_{0} \Vert ^{p}- \int_{\Omega} \vert u_{0} \vert ^{p^{*}}\,dx- \beta \int_{\Omega} \vert u_{0} \vert ^{p} \vert x \vert ^{\alpha-p}\,dx-\lambda \int_{\Omega} \vert u_{0} \vert ^{q}\,dx=0, $$

combining with (2.3), we obtain

2.7 $$ \bigl(p-p^{*}\bigr) \Vert u_{0} \Vert ^{p}=\bigl(p-p^{*}\bigr)\beta \int_{\Omega} \vert u_{0} \vert ^{p} \vert x \vert ^{\alpha-p}\,dx +\bigl(p^{*}-q\bigr)\lambda \int_{\Omega} \vert u_{0} \vert ^{q}\,dx. $$

Equations (2.6) and (2.7) imply that

$$(p-q) \Vert u_{0} \Vert ^{p}-(p-q)\beta \int_{\Omega} \vert u_{0} \vert ^{p} \vert x \vert ^{\alpha-p}\,dx= \bigl(p^{*}-q\bigr) \int_{\Omega} \vert u_{0} \vert ^{p^{*}}\,dx, $$

that is,

2.8 $$ \int_{\Omega} \vert u_{0} \vert ^{p^{*}}\,dx \geq\frac{p-q}{p^{*}-q} \biggl(1-\frac{\beta}{\beta_{1}}\biggr) \Vert u_{0} \Vert ^{p}. $$

Similarly,

$$\bigl(p-p^{*}\bigr) \Vert u_{0} \Vert ^{p}- \bigl(p-p^{*}\bigr)\beta \int_{\Omega } \vert u_{0} \vert ^{p} \vert x \vert ^{\alpha-p}\,dx =\lambda\bigl(q-p^{*}\bigr) \int_{\Omega} \vert u_{0} \vert ^{q}\,dx, $$

that is,

2.9 $$ \lambda \int_{\Omega} \vert u_{0} \vert ^{q}\,dx \geq\frac{p-p^{*}}{q-p^{*}}\biggl(1-\frac {\beta}{\beta_{1}}\biggr) \Vert u_{0} \Vert ^{p}. $$

Note that (1.3) holds for $u\in\mathcal{N}_{\lambda}^{0}\backslash\{0\} $. Then

$$\begin{aligned} \Theta&:=\frac{( \vert \Omega \vert ^{\frac{p^{*} -q}{p^{*}}})^{\frac {p-p^{*}}{q-p}}}{S_{\mu,0}^{\frac{q-p^{*}}{q-p}}} \frac{ \Vert u_{0} \Vert ^{\frac{p(q-p^{*})}{q-p}}}{(\int_{\Omega}(u_{0}^{+})^{q} \,dx)^{\frac {p-p^{*}}{q-p}}}- \int_{\Omega} \vert u_{0} \vert ^{p^{*}}\,dx \\ &>\biggl[\frac{1}{S_{\mu,0}^{\frac{q-p^{*}}{q-p}}}\biggl(\frac{ \Vert u_{0} \Vert ^{p}}{(\int _{\Omega} \vert u_{0} \vert ^{p^{*}}\,dx)^{\frac{p}{p^{*}}}}\biggr)^{\frac{q-p^{*}}{q-p}}-1 \biggr] \int _{\Omega} \vert u_{0} \vert ^{p^{*}}\,dx \geq0. \end{aligned}$$

It follows from (2.8) and (2.9) that

$$\begin{aligned} \Theta&=\frac{( \vert \Omega \vert ^{\frac{p^{*} -q}{p^{*}}})^{\frac {p-p^{*}}{q-p}}}{S_{\mu,0}^{\frac{q-p^{*}}{q-p}}} \lambda^{\frac{p-p^{*}}{q-p}}\frac{ \Vert u_{0} \Vert ^{\frac {p(q-p^{*})}{q-p}}}{(\lambda\int_{\Omega}(u_{0}^{+})^{q} \,dx)^{\frac {p-p^{*}}{q-p}}}- \int_{\Omega} \vert u_{0} \vert ^{p^{*}}\,dx \\ &\leq\frac{( \vert \Omega \vert ^{\frac{p^{*} -q}{p^{*}}})^{\frac{p-p^{*}}{q-p}}}{S_{\mu ,0}^{\frac{q-p^{*}}{q-p}}} \lambda^{\frac{p-p^{*}}{q-p}}\frac{ \Vert u_{0} \Vert ^{p}}{[(\frac {p-p^{*}}{q-p^{*}})(1-\frac{\beta}{\beta_{1}})]^{\frac{p-p^{*}}{q-p}}}-\biggl( \frac {p-q}{p^{*} -q}\biggr) \biggl(1-\frac{\beta}{\beta_{1}}\biggr) \Vert u_{0} \Vert ^{p} \\ &=\biggl[\frac{( \vert \Omega \vert ^{\frac{p^{*} -q}{p^{*}}})^{\frac{p-p^{*}}{q-p}}}{S_{\mu ,0}^{\frac{q-p^{*}}{q-p}}} \frac{\lambda^{\frac{p-p^{*}}{q-p}}}{[(\frac{p-p^{*}}{q-p^{*}})(1-\frac{\beta }{\beta_{1}})]^{\frac{p-p^{*}}{q-p}}}-\biggl(\frac{p-q}{p^{*} -q}\biggr) \biggl(1-\frac{\beta }{\beta_{1}}\biggr)\biggr] \Vert u_{0} \Vert ^{p} \\ &< 0, \end{aligned}$$

for $0<\lambda<T_{1}$. This is a contradiction. □

### Lemma 2.2

$I_{\lambda}$ *is coercive and bounded below on* $\mathcal{N}_{\lambda}$.

### Proof

For $u\in\mathcal{N}_{\lambda}$, we can deduce from (1.3) and (2.5) that

$$\begin{aligned} I_{\lambda}(u)&=\frac{1}{p} \Vert u \Vert ^{p}- \frac{\beta}{p} \int_{\Omega} \vert u \vert ^{p} \vert x \vert ^{\alpha-p}\,dx- \frac{1}{p^{*}} \int_{\Omega} \vert u \vert ^{p^{*}}\,dx - \frac{\lambda}{q} \int_{\Omega} \vert u \vert ^{q}\,dx \\ &=\biggl(\frac{1}{p}-\frac{1}{p^{*}}\biggr) \Vert u \Vert ^{p} -\biggl(\frac{1}{p}-\frac{1}{p^{*}}\biggr)\beta \int_{\Omega} \vert u \vert ^{p} \vert x \vert ^{\alpha-p}\,dx-\biggl(\frac{1}{q}-\frac{1}{p^{*}}\biggr)\lambda \int_{\Omega} \vert u \vert ^{q}\,dx \\ &\geq\biggl(\frac{1}{p}-\frac{1}{p^{*}}\biggr) \biggl(1- \frac{\beta}{\beta_{1}}\biggr) \Vert u \Vert ^{p}-\lambda \biggl( \frac{1}{q}-\frac{1}{p^{*}}\biggr) \vert \Omega \vert ^{\frac{p^{*} -q}{p^{*}}}S_{\mu,0}^{-\frac{q}{p}} \Vert u \Vert ^{q}. \end{aligned}$$

Note that $1< q< p$ and $0<\beta<\beta_{1}$, we see that $I_{\lambda}$ is coercive and bounded below on $\mathcal{N}_{\lambda}$. □

From Lemma 2.1, we know that $\mathcal{N}_{\lambda}^{+}$ and $\mathcal {N}_{\lambda}^{-}$ are nonempty. Furthermore, taking into account Lemma 2.2, we define

$$\kappa_{\lambda}=\inf_{u\in\mathcal{N}_{\lambda}}I_{\lambda}(u), \qquad\kappa _{\lambda}^{+}=\inf_{u\in\mathcal{N}_{\lambda}^{+}}I_{\lambda}(u),\qquad \kappa_{\lambda}^{-}=\inf_{u\in\mathcal{N}_{\lambda}^{-}}I_{\lambda }(u). $$

### Lemma 2.3

$\kappa_{\lambda}\leq\kappa_{\lambda}^{+}<0$.

### Proof

For $u\in\mathcal{N}_{{\lambda}}^{+}$, using (2.1) and (2.2), we have

$$(p-q) \Vert u \Vert ^{p}-(p-q)\beta \int_{\Omega} \vert u \vert ^{p} \vert x \vert ^{\alpha-p}\,dx >\bigl(p^{*}-q\bigr) \int_{\Omega} \vert u \vert ^{p^{*}}\,dx $$

and

$$(p-q) \Vert u \Vert ^{p}\biggl(1-\frac{\beta}{\beta_{1}}\biggr)> \bigl(p^{*}-q\bigr) \int_{\Omega} \vert u \vert ^{p^{*}}\,dx, $$

that is,

2.10 $$ \int_{\Omega} \vert u \vert ^{p^{*}}\,dx< \frac{p-q}{p^{*}-q}\biggl(1-\frac{\beta}{\beta _{1}}\biggr) \Vert u \Vert ^{p}. $$

By (2.10), we get

$$\begin{aligned} I_{\lambda}(u)&=\biggl(\frac{1}{p}-\frac{1}{q}\biggr) \Vert u \Vert ^{p}- \biggl(\frac{1}{p}-\frac{1}{q}\biggr) \beta \int_{\Omega} \vert u \vert ^{p} \vert x \vert ^{\alpha-p}\,dx -\biggl(\frac{1}{p^{*}}-\frac{1}{q}\biggr) \int_{\Omega} \vert u \vert ^{p^{*}}\,dx \\ &< \biggl(\frac{1}{p}-\frac{1}{q}\biggr) \biggl(1- \frac{\beta}{\beta_{1}}\biggr) \Vert u \Vert ^{p}-\biggl( \frac{1}{p^{*}}-\frac{1}{q}\biggr) \biggl(1-\frac{\beta}{\beta_{1}}\biggr) \biggl(\frac{p-q}{p^{*}-q}\biggr) \Vert u \Vert ^{p} \\ &=\biggl(1-\frac{\beta}{\beta_{1}}\biggr) (q-p) \biggl(\frac{1}{qp} - \frac{1}{qp^{*}}\biggr) \Vert u \Vert ^{p} \\ &< 0. \end{aligned}$$

Therefore, we have $\kappa_{\lambda}\leq\kappa_{\lambda}^{+}<0$. □

### Lemma 2.4

*For* $u\in\mathcal{N}_{\lambda}$, *there exist* $\varepsilon>0$ *and a differentiable function* $\widehat {f}=\widehat{f}(\omega): B(0,\varepsilon)\subset W_{0}^{1,p}(\Omega )\longrightarrow\mathbb{R}^{+}$ *such that*

$$\widehat{f}(0)=1,\qquad \widehat{f}(\omega) (u+\omega)\in\mathcal{N}_{\lambda },\quad \forall\omega\in B(0,\varepsilon). $$

### Proof

Define

$$\widehat{F}:\mathbb{R}\times W_{0}^{1,p}(\Omega) \longrightarrow\mathbb{R} $$

as follows:

$$\begin{aligned} \widehat{F}(s,\omega)={}&s^{p-q} \int_{\Omega} \biggl( \bigl\vert \nabla(u+\omega) \bigr\vert ^{p}-\mu\frac{ \vert u+\omega \vert ^{p}}{ \vert x \vert ^{p}}\biggr)\,dx -s^{p-q}\beta \int_{\Omega} \vert u+\omega \vert ^{p} \vert x \vert ^{\alpha-p}\,dx \\ & {}-s^{p^{*}-q} \int_{\Omega} \vert u+\omega \vert ^{p^{*}}\,dx -\lambda \int_{\Omega} \vert u+\omega \vert ^{q}\,dx, \quad u\in \mathcal{N}_{\lambda}. \end{aligned}$$

It is clear that

$$\widehat{F}(1,0)= \int_{\Omega}\biggl( \vert \nabla u \vert ^{p}-\mu \frac { \vert u \vert ^{p}}{ \vert x \vert ^{p}}\biggr)\,dx-\beta \int_{\Omega} \vert u \vert ^{p} \vert x \vert ^{\alpha-p}\,dx- \int_{\Omega} \vert u \vert ^{p^{*}}\,dx-\lambda \int_{\Omega} \vert u \vert ^{q}\,dx $$

and

$$\begin{aligned} \widehat{F}_{s}(s,\omega)={}&(p-q)s^{p-q-1} \int_{\Omega}\biggl( \bigl\vert \nabla(u+\omega) \bigr\vert ^{p}- \mu\frac{ \vert u+\omega \vert ^{p}}{ \vert x \vert ^{p}}\biggr)\,dx\\ &{} -(p-q)s^{p-q-1}\beta \int_{\Omega} \vert u+\omega \vert ^{p} \vert x \vert ^{\alpha-p}\,dx \\ &{}-\bigl(p^{*}-q\bigr)s^{p^{*}-q-1} \int_{\Omega} \vert u+\omega \vert ^{p^{*}}\,dx, \end{aligned}$$

which implies that

$$\begin{aligned} \widehat{F}_{s}(1,0)={}&(p-q) \int_{\Omega}\biggl( \vert \nabla u \vert ^{p}-\mu \frac{ \vert u \vert ^{p}}{ \vert x \vert ^{p}}\biggr)\,dx-(p-q)\beta \int_{\Omega} \vert u \vert ^{p} \vert x \vert ^{\alpha-p}\,dx \\ &{}-\bigl(p^{*}-q\bigr) \int_{\Omega} \vert u \vert ^{p^{*}}\,dx. \end{aligned}$$

Lemma 2.1 tells us that $\widehat{F}_{s}(1,0)\neq0$. Thus, by the implicit function theorem at the point $(0,1)$, there exist $\varepsilon >0$, and a differentiable function

$$\widehat{f}:B(0,\varepsilon)\subset W_{0}^{1,p}(\Omega) \longrightarrow \mathbb{R}^{+} $$

such that

$$\widehat{f}(0)=1,\qquad \widehat{f}(\omega)>0\quad \mbox{and} \quad\widehat{f}(\omega ) (u+\omega)\in \mathcal{N}_{\lambda}, \quad\forall\omega\in B(0,\varepsilon). $$

 □

### Lemma 2.5

*For* $u\in\mathcal{N}_{\lambda}^{-}$, *there exist* $\varepsilon>0$ *and a differentiable function* $\widetilde {f}=\widetilde{f}(v): B(0,\varepsilon)\subset W_{0}^{1,p}(\Omega )\longrightarrow\mathbb{R}^{+}$ *such that*

$$\widetilde{f}(0)=1\quad \textit{and} \quad\widetilde{f}(v) (u+v)\in\mathcal{N}_{\lambda }^{-},\quad \forall v\in B(0, \varepsilon). $$

### Proof

The proof is similar to that of Lemma 2.4, and we omit it here. □

### Lemma 2.6

*If* $\{u_{n}\}\subset\mathcal {N}_{\lambda}$ *is a minimizing sequence of* $I_{\lambda}$, *for every* $\phi \in W_{0}^{1,p}(\Omega)$, *then*

2.11 $$ -\frac{ \vert f_{n}^{\prime}(0) \vert \Vert u_{n} \Vert + \Vert \phi \Vert }{n}\leq\bigl\langle I_{\lambda }^{\prime}(u_{n}), \phi\bigr\rangle \leq\frac{ \vert f_{n}^{\prime}(0) \vert \Vert u_{n} \Vert + \Vert \phi \Vert }{n}. $$

### Proof

It follows from Lemma 2.2 that $I_{\lambda}$ is coercive on $\mathcal{N}_{\lambda}$. Using the Ekeland variational principle [19], we can find a minimizing sequence $\{u_{n}\}\subset \mathcal{N}_{\lambda}$ of $I_{\lambda}$ satisfying

2.12 $$ I_{\lambda}(u_{n})< \kappa_{\lambda}+ \frac{1}{n},\qquad I_{\lambda}(u_{n})\leq I_{\lambda}(w)+ \frac{1}{n} \Vert w-u_{n} \Vert \quad \forall w\in \mathcal{N}_{\lambda}. $$

Without loss of generality, we can assume that $u_{n}\geq0$. By Lemma 2.2, we know that $\{u_{n}\}$ is bounded in $W_{0}^{1,p}(\Omega)$. As a consequence, there exist a subsequence (still denoted by $\{u_{n}\}$) and $u_{*}$ in $W_{0}^{1,p}(\Omega)$ such that

2.13 $$ \textstyle\begin{cases} u_{n}\rightharpoonup u_{*}\quad \mbox{weakly in }W_{0}^{1,p}(\Omega), \\ u_{n}\rightarrow u_{*}\quad \mbox{strongly in }L^{p}(\Omega)\ (1\leq p< p^{*}), \\ u_{n}(x)\rightarrow u_{*}(x)\quad \mbox{a.e. in }\Omega. \end{cases} $$

From Lemma 2.4, for $s>0$ sufficiently small and $\phi\in W_{0}^{1,p}(\Omega)$, and set $u=u_{n}$, $\omega=s\phi\in W_{0}^{1,p}(\Omega)$, we can find that $f_{n}(s)=f_{n}(s\phi)$ such that $f_{n}(0)=1$ and $f_{n}(s)(u_{n}+s\phi)\in\mathcal{N}_{\lambda}$. Since

2.14 $$ \Vert u_{n} \Vert ^{p}- \int_{\Omega} \vert u_{n} \vert ^{p^{*}}\,dx- \beta \int_{\Omega } \vert u_{n} \vert ^{p} \vert x \vert ^{\alpha-p}\,dx -\lambda \int_{\Omega} \vert u_{n} \vert ^{q}\,dx=0. $$

By (2.12), we obtain

2.15 $$\begin{aligned} \frac{1}{n}\bigl[ \bigl\vert f_{n}(s)-1 \bigr\vert \Vert u_{n} \Vert +sf_{n}(s) \Vert \phi \Vert \bigr]&\geq\frac{1}{n} \bigl\Vert f_{n}(s) (u_{n}+s\phi)-u_{n} \bigr\Vert \\ &\geq I_{\lambda}(u_{n})-I_{\lambda }\bigl[f_{n}(s) (u_{n}+s\phi)\bigr]. \end{aligned}$$

Notice that

$$\begin{aligned} I_{\lambda}\bigl[f_{n}(s) (u_{n}+s\phi)\bigr]={}& \frac{1}{p} \bigl\Vert f_{n}(s) (u_{n}+s\phi) \bigr\Vert ^{p} -\frac{\beta}{p} \int_{\Omega} \vert x \vert ^{\alpha-p} \bigl\vert f_{n}(s) (u_{n}+s\phi) \bigr\vert ^{p}\,dx \\ &{}-\frac{1}{p^{*}} \int_{\Omega} \bigl\vert f_{n}(s) (u_{n}+s\phi) \bigr\vert ^{p^{*}}\,dx-\frac{\lambda}{q} \int_{\Omega} \bigl\vert f_{n}(s) (u_{n}+s\phi) \bigr\vert ^{q}\,dx \\ ={}&\frac{f_{n}^{p}(s)}{p} \Vert u_{n}+s\phi \Vert ^{p}- \frac{\beta}{p} f_{n}^{p}(s) \int_{\Omega} \vert x \vert ^{\alpha-p} \bigl\vert (u_{n}+s\phi) \bigr\vert ^{p}\,dx \\ &{}-\frac{f_{n}^{p^{*}}(s)}{p^{*}} \int_{\Omega} \bigl\vert (u_{n}+s\phi) \bigr\vert ^{p^{*}}\,dx- \frac{\lambda}{q}f_{n}^{q}(s) \int_{\Omega} \bigl\vert (u_{n}+s\phi) \bigr\vert ^{q}\,dx. \end{aligned}$$

Therefore

$$\begin{aligned} &I_{\lambda}(u_{n})-I_{\lambda}\bigl[f_{n}(s) (u_{n}+s\phi )\bigr] \\ &\quad=\frac{1}{p} \Vert u_{n} \Vert ^{p}- \frac{f_{n}^{p}(s)}{p} \Vert u_{n} \Vert ^{p} + \frac{f_{n}^{p^{*}}(s)}{p^{*}} \int_{\Omega} \vert u_{n}+s\phi \vert ^{p^{*}}\,dx -\frac{1}{p^{*}} \int_{\Omega} \vert u_{n}+s\phi \vert ^{p^{*}}\,dx \\ &\qquad{}+\frac{\lambda}{q}f_{n}^{q}(s) \int_{\Omega} \vert u_{n}+s\phi \vert ^{q}\,dx -\frac{\lambda}{q} \int_{\Omega} \vert u_{n}+s\phi \vert ^{q}\,dx+\frac{\beta}{p} f_{n}^{p}(s) \int_{\Omega} \vert x \vert ^{\alpha-p} \vert u_{n}+s\phi \vert ^{p}\,dx \\ &\qquad{}-\frac{\beta}{p} \int_{\Omega} \vert x \vert ^{\alpha-p} \vert u_{n}+s\phi \vert ^{p}\,dx +\frac{f_{n}^{p}(s)}{p} \Vert u_{n} \Vert ^{p}-\frac{f_{n}^{p}(s)}{p} \Vert u_{n}+s\phi \Vert ^{p} +\frac{1}{p^{*}} \int_{\Omega} \vert u_{n}+s\phi \vert ^{p^{*}}\,dx \\ &\qquad{}-\frac{1}{p^{*}} \int_{\Omega} \vert u_{n} \vert ^{p^{*}}\,dx +\frac{\lambda}{q} \int_{\Omega} \vert u_{n}+s\phi \vert ^{q}\,dx -\frac{\lambda}{q} \int_{\Omega} \vert u_{n} \vert ^{q}\,dx \\ &\qquad{}+\frac{\beta}{p} \int_{\Omega} \vert x \vert ^{\alpha-p} \vert u_{n}+s\phi \vert ^{p}\,dx-\frac{\beta}{p} \int_{\Omega} \vert u_{n} \vert ^{p} \vert x \vert ^{\alpha-p}\,dx \\ &\quad=\frac{1-f_{n}^{p}(s)}{p} \Vert u_{n} \Vert ^{p}+ \frac{f_{n}^{p^{*}}(s)-1}{p^{*}} \int_{\Omega} \vert u_{n}+s\phi \vert ^{p^{*}}\,dx+\frac{\lambda}{q}\bigl(f_{n}^{q}(s)-1 \bigr) \int_{\Omega} \vert u_{n}+s\phi \vert ^{q}\,dx \\ &\qquad{}+\frac{\beta}{p}\bigl(f_{n}^{p}(s)-1\bigr) \int_{\Omega} \vert x \vert ^{\alpha-p} \vert u_{n}+s\phi \vert ^{p}\,dx+\frac{f_{n}^{p}(s)}{p}\bigl( \Vert u_{n} \Vert ^{p}- \Vert u_{n}+s\phi \Vert ^{p}\bigr) \\ &\qquad{}+\frac{1}{p^{*}}\biggl( \int_{\Omega} \vert u_{n}+s\phi \vert ^{p^{*}}\,dx- \int_{\Omega} \vert u_{n} \vert ^{p^{*}}\,dx \biggr)+\frac{\lambda}{q} \int_{\Omega}\bigl( \vert u_{n}+s\phi \vert ^{q}- \vert u_{n} \vert ^{q}\bigr)\,dx \\ &\qquad{}+\frac{\beta}{p} \int_{\Omega}\bigl[ \vert u_{n}+s\phi \vert ^{p} - \vert u_{n} \vert ^{p}\bigr] \vert x \vert ^{\alpha-p}\,dx. \end{aligned}$$

Dividing by $s>0$ and taking the limit for $s\rightarrow0$, combining with (2.14) and (2.15), we have

$$\begin{aligned} &\frac{ \vert f_{n}^{\prime}(0) \vert \Vert u_{n} \Vert + \Vert \phi \Vert }{n} \\ &\quad\geq-f_{n}^{\prime}(0) \Vert u_{n} \Vert ^{p}+f_{n}^{\prime}(0) \int_{\Omega} \vert u_{n} \vert ^{p^{*}}\,dx +\lambda f_{n}^{\prime}(0) \int_{\Omega} \vert u_{n} \vert ^{q}\,dx \\ &\qquad{}+\beta f_{n}^{\prime}(0) \int_{\Omega} \vert u_{n} \vert ^{p} \vert x \vert ^{\alpha-p}\,dx- \int_{\Omega} \vert \nabla u_{n} \vert ^{p-2}\nabla u_{n}\nabla\phi \,dx\\ &\qquad{}+\mu \int_{\Omega}\frac{ \vert u_{n} \vert ^{p-2}u_{n}\phi}{ \vert x \vert ^{p}}\,dx + \int_{\Omega} \vert u_{n} \vert ^{p^{*}-1} \phi \,dx \\ &\qquad{}+\lambda \int_{\Omega} \vert u_{n} \vert ^{q-1} \phi \,dx+ \beta \int_{\Omega} \vert u_{n} \vert ^{p-1} \phi \vert x \vert ^{\alpha-p}\,dx \\ &\quad=-f_{n}^{\prime}(0)\biggl[ \Vert u_{n} \Vert ^{p}- \int_{\Omega} \vert u_{n} \vert ^{p^{*}}\,dx- \lambda \int_{\Omega} \vert u_{n} \vert ^{q}\,dx- \beta \int_{\Omega} \vert u_{n} \vert ^{p} \vert x \vert ^{\alpha-p}\,dx\biggr] -\bigl\langle I_{\lambda}^{\prime}, \phi\bigr\rangle \\ &\quad=-\bigl\langle I_{\lambda}^{\prime},\phi\bigr\rangle . \end{aligned}$$

Consequently

2.16 $$ -\frac{ \vert f_{n}^{\prime}(0) \vert \Vert u_{n} \Vert + \Vert \phi \Vert }{n}\leq\bigl\langle I_{\lambda }^{\prime}, \phi\bigr\rangle $$

for every $\phi\in W_{0}^{1,p}(\Omega)$. Note that (2.16) holds equally for −*ϕ*, we see that (2.11) holds. □

### Lemma 2.7

see [8, 10]

*Set* $D^{1,p}(\mathbb {R}^{N})=\{u\in L^{p^{*}}(\mathbb{R}^{N}): \vert \nabla u \vert \in L^{p} (\mathbb{R}^{N})\}$. *Assume that* $1< p< N$ *and* $0\leq\mu<\overline{\mu}$. *Then the limiting problem*

2.17 $$ \textstyle\begin{cases} -\Delta_{p}u-\mu\frac{u^{p-1}}{ \vert x \vert ^{p}}=u^{p^{*}-1}\quad \textit{in }\mathbb {R}^{N}\backslash\{0\}, \\ u>0\quad \textit{in }\mathbb{R}^{N}\backslash\{0\},\\ u\in D^{1,p}(\mathbb{R}^{N}) \end{cases} $$

*has radially symmetric ground states*

$$V_{\epsilon}(x)=\epsilon^{\frac{p-N}{p}}U_{p,\mu}\biggl( \frac{x}{\epsilon }\biggr)=\epsilon^{\frac{p-N}{p}}U_{p,\mu}\biggl( \frac{ \vert x \vert }{\epsilon}\biggr)\quad \forall \epsilon>0, $$

*such that*

$$\int_{\mathbb{R}^{N}}\biggl( \bigl\vert \nabla V_{\epsilon}(x) \bigr\vert ^{p}-\mu\frac{ \vert V_{\epsilon }(x) \vert ^{p}}{ \vert x \vert ^{p}}\biggr)\,dx = \int_{\mathbb{R}^{N}} \bigl\vert V_{\epsilon}(x) \bigr\vert ^{p^{*}}\,dx=S_{\mu,0}^{\frac{N}{p}}, $$

*where the function* $U_{p,\mu}(x)=U_{p,\mu}( \vert x \vert )$ *is the unique radial solution of the above limiting problem with*

$$U_{p,\mu}(1)=\biggl(\frac{N(\overline{\mu}-\mu)}{N-p}\biggr)^{\frac{1}{p^{*}-p}}. $$

In the following, we define $\Lambda=\frac{1}{N}S_{\mu,0}^{\frac{N}{p}}$.

### Lemma 2.8

*Let* $\{u_{n}\}\subset\mathcal {N}^{-}_{\lambda}$ *be a minimizing sequence for* $I_{\lambda}$ *with* $\kappa_{\lambda}^{-}<\Lambda-D\lambda^{\frac{p}{p-q}}$, *where*

2.18 $$ D=\frac{p-q}{p}\biggl[\frac{p^{*} -q}{p^{*} q} \vert \Omega \vert ^{\frac{p^{*}-q}{p^{*}}} S_{\mu,0}^{-\frac{q}{p}}\biggl(\frac{\beta_{1} -\beta}{N \beta_{1}} \biggr)^{-\frac {q}{p}}\biggr]^{\frac{p}{p-q}}. $$

*Then there exists* $u\in W_{0}^{1,p}(\Omega)$ *such that* $u_{n}\rightarrow u$ *in* $L^{p^{*}}(\Omega)$.

### Proof

Since

2.19 $$ I_{\lambda}(u_{n})\rightarrow \kappa_{\lambda}^{-}\quad \mbox{as }n\rightarrow+\infty. $$

By Lemma 2.2, we know that $\{u_{n}\}$ is bounded in $W_{0}^{1,p}(\Omega )$. In fact, we can deduce from (1.3) and (2.19) that

$$\begin{aligned} &1+\kappa_{\lambda}^{-}+o\bigl( \Vert u_{n} \Vert \bigr) \\ &\quad\geq I_{\lambda }(u_{n})-\frac{1}{p^{*}}\bigl\langle I_{\lambda}^{\prime}(u_{n}),u_{n}\bigr\rangle \\ &\quad=\frac{1}{p} \Vert u_{n} \Vert ^{p}- \frac{\beta}{p} \int_{\Omega} \vert u_{n} \vert ^{p} \vert x \vert ^{\alpha-p}\,dx -\frac{1}{p^{*}} \int_{\Omega} \vert u_{n} \vert ^{p^{*}}\,dx -\frac{\lambda}{q} \int_{\Omega} \vert u_{n} \vert ^{q}\,dx \\ &\qquad{}-\frac{1}{p^{*}}\biggl( \Vert u_{n} \Vert ^{p} - \int_{\Omega} \vert u_{n} \vert ^{p^{*}}\,dx- \lambda \int_{\Omega} \vert u_{n} \vert ^{q}\,dx- \beta \int_{\Omega} \vert u_{n} \vert ^{p} \vert x \vert ^{\alpha-p}\,dx\biggr) \\ &\quad=\biggl(\frac{1}{p}-\frac{1}{p^{*}}\biggr) \Vert u_{n} \Vert ^{p}-\biggl(\frac{1}{p} -\frac{1}{p^{*}}\biggr) \beta \int_{\Omega} \vert u_{n} \vert ^{p} \vert x \vert ^{\alpha-p}\,dx\\ &\qquad{} +\biggl(\frac{1}{p^{*}}-\frac{1}{q} \biggr)\lambda \int_{\Omega} \vert u_{n} \vert ^{q}\,dx \\ &\quad \geq\biggl(\frac{1}{p}-\frac{1}{p^{*}}\biggr) \biggl(1- \frac{\beta}{ \beta_{1}}\biggr) \Vert u_{n} \Vert ^{p} + \biggl(\frac{1}{p^{*}}-\frac{1}{q}\biggr)\lambda \int_{\Omega} \vert u_{n} \vert ^{q}\,dx \\ &\quad\geq \biggl(\frac{1}{p}-\frac{1}{p^{*}}\biggr) \biggl(1- \frac{\beta}{\beta_{1}}\biggr) \Vert u_{n} \Vert ^{p}\\ &\qquad{} + \biggl(\frac{1}{p^{*}}-\frac{1}{q}\biggr)\lambda \vert \Omega \vert ^{\frac{p^{*}-q}{p^{*}}}\biggl( \int_{\Omega} \vert u_{n} \vert ^{p^{*}}\,dx \biggr)^{\frac{q}{p^{*}}} \\ &\quad\geq \biggl(\frac{1}{p}-\frac{1}{p^{*}}\biggr) \biggl(1- \frac{\beta}{\beta_{1}}\biggr) \Vert u_{n} \Vert ^{p} + \biggl(\frac{1}{p^{*}}-\frac{1}{q}\biggr)\lambda \vert \Omega \vert ^{\frac {p^{*}-q}{p^{*}}}S_{\mu,0}^{-\frac{q}{p}} \Vert u_{n} \Vert ^{q}, \end{aligned}$$

where $0<\beta<\beta_{1}$, $1< q< p$, we see that $\{u_{n}\}$ is bounded in $W_{0}^{1,p}(\Omega)$. We can choose a subsequence (still denoted by $\{u_{n}\}$) and $u\in W_{0}^{1,p}(\Omega)$ satisfying

2.20 $$ \textstyle\begin{cases} u_{n}\rightharpoonup u\quad \mbox{weakly in }W_{0}^{1,p}(\Omega), \\ u_{n}\rightarrow u \quad\mbox{strongly in }L^{p}(\Omega)\ (1\leq p< p^{*}). \\ u_{n}(x)\rightarrow u(x) \quad\mbox{a.e. in }\Omega. \end{cases} $$

In term of the concentration compactness principle, going if necessary to a subsequence, there exist an at most countable set $\mathcal{J}$, a set of points $\{x_{j}\}_{j\in\mathcal{J}}\subset\Omega\setminus\{0\} $, and real numbers $\mu_{j}$, $\nu_{j}$, $\widetilde{\chi_{0}}$ such that

$$\begin{aligned} &\vert \nabla u_{n} \vert ^{p}\rightharpoonup \,d\mu\geq \vert \nabla u \vert ^{p}+\sum_{j\in \mathcal{J}} \mu_{j}\delta_{x_{j}}+\mu_{0}\delta_{0}, \\ &\vert u_{n} \vert ^{p^{*}}\rightharpoonup \,d\nu= \vert u \vert ^{p^{*}}+\sum_{j\in\mathcal {J}} \nu_{j}\delta_{x_{j}}+\nu_{0}\delta_{0}, \\ &\frac{ \vert u_{n} \vert ^{p}}{ \vert x \vert ^{p}}\rightharpoonup \,d\widetilde{\chi}=\frac { \vert u \vert ^{p}}{ \vert x \vert ^{p}}+ \widetilde{\chi_{0}}\delta_{0}, \end{aligned}$$

where $\delta_{x_{j}}$ is the Dirac mass at $x_{j}$.

Let *ϵ* be sufficient small satisfying $0\notin B(x_{j}, \epsilon )$ and $B(x_{j}, \epsilon)\cap B(x_{i}, \epsilon)=\emptyset$ for $i\neq j, i, j=1, 2, \ldots, k$. Let $\psi_{\epsilon,j}(x)$ be a smooth cut-off function centered at $x_{j}$ such that $0\leq\psi_{\epsilon ,j}(x)\leq1$, $\psi_{\epsilon,j}(x)=1$ for $x\in B(x_{j}, \frac{\epsilon}{2})$, $\psi_{\epsilon,j}(x)=0$ for $x\in\Omega\backslash B(x_{j},\epsilon)$ and $\vert \nabla\psi_{\epsilon,j}(x) \vert \leq\frac{4}{\epsilon}$. Note that

$$\begin{aligned} &\bigl\langle I_{\lambda}^{\prime}(u_{n}),u_{n} \psi_{\epsilon ,j}(x)\bigr\rangle \\ &\quad= \int_{\Omega} \vert \nabla u_{n} \vert ^{p}\psi_{\epsilon,j}(x)\,dx+ \int_{\Omega}u_{n} \vert \nabla u_{n} \vert ^{p-2}\nabla u_{n}\nabla\psi_{\epsilon,j}(x)\,dx -\mu \int_{\Omega}\frac{ \vert u_{n} \vert ^{p}}{ \vert x \vert ^{p}}\psi_{\epsilon,j}(x)\,dx \\ &\qquad{}- \int_{\Omega} \vert u_{n} \vert ^{p^{*}} \psi_{\epsilon,j}(x)\,dx-\lambda \int_{\Omega} \vert u_{n} \vert ^{q} \psi_{\epsilon,j}(x)\,dx-\beta \int_{\Omega} \vert u_{n} \vert ^{p} \vert x \vert ^{\alpha-p} \psi_{\epsilon,j}(x)\,dx. \end{aligned}$$

Furthermore, we have

$$\begin{aligned} &\lim_{n\rightarrow\infty} \int_{\Omega} \vert \nabla u_{n} \vert ^{p}\psi_{\epsilon,j}(x)\,dx= \int_{\Omega} \psi_{\epsilon,j}(x)\,d\mu\geq \int_{\Omega} \vert \nabla u \vert ^{p} \psi_{\epsilon ,j}(x)\,dx+\mu_{j}, \\ &\lim_{n\rightarrow\infty} \int_{\Omega } \vert u_{n} \vert ^{p^{*}} \psi_{\epsilon,j}(x)\,dx = \int_{\Omega}\psi_{\epsilon,j}(x)\,d\nu= \int_{\Omega} \vert u \vert ^{p^{*}} \psi_{\epsilon,j}(x)\,dx+\nu_{j}, \\ &\lim_{\epsilon\rightarrow0}\lim_{n\rightarrow\infty} \biggl\vert \int_{\Omega }u_{n} \vert \nabla u_{n} \vert ^{p-2}\nabla u_{n}\cdot\nabla\psi_{\epsilon,j}(x) \biggr\vert =0, \\ &\lim_{\epsilon\rightarrow0}\lim_{n\rightarrow \infty} \biggl\vert \int_{\Omega}\frac{ \vert u_{n} \vert ^{p}}{ \vert x \vert ^{p}}\psi_{\epsilon,j} (x) \biggr\vert =0. \end{aligned}$$

By (1.3), we deduce that

$$\begin{aligned} \biggl\vert \int_{\Omega} \vert u_{n} \vert ^{q} \psi_{\epsilon,j}\,dx \biggr\vert &\leq \int _{B(x_{j},\epsilon)} \vert u_{n} \vert ^{q}\,dx \\ &\leq\biggl( \int_{B(x_{j},\epsilon)} \vert u_{n} \vert ^{q\frac {p^{*}}{q}}\,dx \biggr)^{\frac{q}{p^{*}}} \biggl( \int_{B(x_{j},\epsilon)}\,dx\biggr)^{\frac{p^{*}-q}{p^{*}}} \\ &\leq S_{\mu,0}^{-\frac{q}{p}} \Vert u_{n} \Vert ^{q} \biggl( \int_{B(x_{j},\epsilon )}\,dx\biggr)^{\frac{p^{*}-q}{p^{*}}} \\ &\leq S_{\mu,0}^{-\frac{q}{p}}\biggl( \int_{0}^{\epsilon}r^{N-1}\,dr\biggr)^{\frac {p^{*}-q}{p^{*}}} \Vert u_{n} \Vert ^{q} \\ &=\biggl(\frac{1}{N}\biggr)^{\frac{p^{*}-q}{p^{*}}}S_{\mu,0}^{-\frac{q}{p}} \epsilon ^{\frac{N(p^{*}-q)}{p^{*}}} \Vert u_{n} \Vert ^{q} \end{aligned}$$

and

$$\begin{aligned} \biggl\vert \int_{\Omega} \vert u_{n} \vert ^{p} \vert x \vert ^{\alpha-p} \psi_{\epsilon,j}(x)\,dx \biggr\vert &\leq \biggl( \int_{B(x_{j},\epsilon)} \vert u_{n} \vert ^{p\frac{p^{*}}{p}}\,dx \biggr)^{\frac{p}{p^{*}}}\biggl( \int_{B(x_{j},\epsilon)} \vert x \vert ^{\frac{p^{*}(\alpha-p)}{p^{*}-p}}\,dx \biggr)^{\frac{p^{*}-p}{p^{*}}} \\ &\leq\biggl( \int_{B(x_{j},\epsilon)} \vert u_{n} \vert ^{p\frac{p^{*}}{p}}\,dx \biggr)^{\frac{p}{p^{*}}}\biggl( \int_{B(x_{j},\epsilon)} \vert x-x_{j} \vert ^{\frac{p^{*}(\alpha-p)}{p^{*}-p}}\,dx\biggr)^{\frac{p^{*}-p}{p^{*}}} \\ &\leq S_{\mu,0}^{-1} \Vert u_{n} \Vert ^{p}\biggl( \int_{0}^{\epsilon} r^{N-1}r^{\frac{p^{*}(\alpha-p)}{p^{*}-p}}\,dr \biggr)^{\frac{p^{*}-p}{p^{*}}} \\ &=S_{\mu,0}^{-1} \Vert u_{n} \Vert ^{p}\biggl(\frac{p}{N\alpha} \epsilon^{\frac{N\alpha}{p}} \biggr)^{\frac{p^{*}-p}{p^{*}}}. \end{aligned}$$

Since $\{u_{n}\}$ is bounded in $W_{0}^{1,p}(\Omega)$, and $u_{n}\rightharpoonup u$ weakly in $L^{p^{*}}(\Omega)$, we conclude that

$$\lim_{\epsilon\rightarrow0}\lim_{n\rightarrow\infty} \int_{\Omega } \vert u_{n} \vert ^{q} \psi_{\epsilon,j}(x)\,dx=0 $$

and

$$\lim_{\epsilon\rightarrow0}\lim_{n\rightarrow\infty} \int_{\Omega } \vert u_{n} \vert ^{p} \vert x \vert ^{\alpha-p}\psi_{\epsilon,j}(x)\,dx=0. $$

By (2.11), we have

$$\begin{aligned} 0=\lim_{\epsilon\rightarrow0}\lim_{n\rightarrow \infty}\bigl\langle I_{\lambda}^{\prime}(u_{n}),u_{n} \psi_{\epsilon,j}(x)\bigr\rangle \geq\mu_{j}-\nu_{j}. \end{aligned}$$

Since $S_{0,0}\nu_{j}^{\frac{p}{p^{*}}}\leq\mu_{j}$, we have $\mu_{j}=\nu _{j}=0$ or $\mu_{j}\geq(S_{0,0})^{\frac{N}{p}}$.

On the other hand, let $\epsilon>0$ be sufficiently small satisfying $x_{j}\notin B(0, \epsilon)$, $\forall j\in\mathcal{J}$. Let $\psi _{\epsilon,0}(x)$ a smooth cut-off function centered at the origin such that $0\leq\psi_{\epsilon,0}(x)\leq1$, $\psi_{\epsilon,0}(x)=1$ for $\vert x \vert \leq\frac{\epsilon}{2}$, $\psi_{\epsilon,0}(x)=0$ for $\vert x \vert \geq \epsilon$ and $\vert \nabla\psi_{\epsilon,0}(x) \vert \leq\frac{4}{\epsilon}$. Hence, we have

$$\begin{aligned} &\lim_{n\rightarrow\infty} \int_{\Omega} \vert \nabla u_{n} \vert ^{p}\psi _{\epsilon,0}(x)\,dx= \int_{\Omega} \psi_{\epsilon,0}(x)\,d\mu\geq \int_{\Omega} \vert \nabla u \vert ^{p} \psi_{\epsilon ,0}(x)\,dx+\mu_{0}, \\ &\lim_{n\rightarrow\infty} \int_{\Omega } \vert u_{n} \vert ^{p^{*}} \psi_{\epsilon,0}(x)\,dx = \int_{\Omega}\psi_{\epsilon,0}(x)\,d\nu= \int_{\Omega} \vert u \vert ^{p^{*}} \psi_{\epsilon,0}(x)\,dx+\nu_{0}, \\ &\lim_{n\rightarrow\infty} \int_{\Omega} \frac{ \vert u_{n} \vert ^{p}}{ \vert x \vert ^{p}}\psi_{\epsilon,0}(x)\,dx= \int_{\Omega}\psi_{\epsilon,0}(x)\,d\widetilde{\chi}= \int_{\Omega} \frac{ \vert u \vert ^{p}}{ \vert x \vert ^{p}}\psi_{\epsilon,0}(x)\,dx+ \widetilde{\chi_{0}}, \\ &\lim_{\epsilon\rightarrow0}\lim_{n\rightarrow\infty} \biggl\vert \int_{\Omega }u_{n} \vert \nabla u_{n} \vert ^{p-2}\nabla u_{n}\cdot\nabla\psi_{\epsilon,0}(x)\,dx \biggr\vert =0, \\ &\lim_{\epsilon\rightarrow0}\lim_{n\rightarrow\infty} \int_{\Omega } \vert u_{n} \vert ^{q} \psi_{\epsilon,0}(x)\,dx=0 \end{aligned}$$

and

$$\lim_{\epsilon\rightarrow0}\lim_{n\rightarrow\infty} \int_{\Omega } \vert u_{n} \vert ^{p} \vert x \vert ^{\alpha-p}\psi_{\epsilon,0}(x)\,dx=0. $$

Therefore

$$0=\lim_{\epsilon\rightarrow0}\lim_{n\rightarrow \infty}\bigl\langle I_{\lambda}^{\prime}(u_{n}),u_{n} \psi_{\epsilon,0}(x)\bigr\rangle \geq\mu_{0}-\mu\widetilde{ \chi_{0}}-\nu_{0}. $$

Combining the definition of $S_{\mu,0}$, we get that $S_{\mu,0}\nu _{0}^{\frac{p}{p^{*}}}\leq\mu_{0}-\mu\widetilde{\chi_{0}}\leq\nu_{0}$, which implies that $\nu_{0}=0$ or $\nu_{0}\geq(S_{\mu,0})^{\frac {N}{p}}$. Now, we prove that $\mu_{j}\geq(S_{0,0})^{\frac{N}{p}}$ and $\nu_{0}\geq(S_{\mu,0})^{\frac{N}{p}}$ are not true. If not, we have

$$\begin{aligned} \kappa_{\lambda}^{-}&=\lim_{n\rightarrow\infty } \biggl[I_{\lambda}(u_{n})-\frac{1}{p^{*}}\bigl\langle I_{\lambda }^{\prime}(u_{n}),u_{n}\bigr\rangle \biggr] \\ &\geq\lim_{n\rightarrow\infty}\biggl[ \biggl(\frac{1}{p}- \frac{1}{p^{*}}\biggr) \Vert u_{n} \Vert ^{p} + \biggl(\frac{1}{p^{*}}-\frac{1}{q}\biggr)\lambda \vert \Omega \vert ^{\frac {p^{*}-q}{p^{*}}}S_{\mu,0}^{-\frac{q}{p}} \Vert u_{n} \Vert ^{q}\biggr] \\ &=\lim_{n\rightarrow\infty}\biggl[\frac{1}{N} \Vert u_{n} \Vert ^{p} +\biggl(\frac{1}{p^{*}}- \frac{1}{q}\biggr)\lambda \vert \Omega \vert ^{\frac {p^{*}-q}{p^{*}}}S_{\mu,0}^{-\frac{q}{p}} \Vert u_{n} \Vert ^{q}\biggr] \\ &\geq\frac{1}{N}\biggl( \Vert u \Vert ^{p}+\sum _{j\in\mathcal{J}}\mu_{j} +\mu_{0}-\mu \widetilde{ \chi_{0}}\biggr) +\biggl(\frac{1}{p^{*}}-\frac{1}{q}\biggr) \lambda \vert \Omega \vert ^{\frac {p^{*}-q}{p^{*}}}S_{\mu,0}^{-\frac{q}{p}} \Vert u \Vert ^{q} \\ &\geq\frac{1}{N}S_{\mu,0}^{\frac{N}{p}}+\frac{1}{N} \Vert u \Vert ^{p} +\biggl(\frac{1}{p^{*}}-\frac{1}{q} \biggr)\lambda \vert \Omega \vert ^{\frac {p^{*}-q}{p^{*}}}S_{\mu,0}^{-\frac{q}{p}} \Vert u \Vert ^{q} \\ &= \frac{1}{N}S_{\mu,0}^{\frac{N}{p}}+\frac{1}{N} \Vert u \Vert ^{p} -\frac{p^{*} -q}{p^{*} q}\lambda \vert \Omega \vert ^{\frac{p^{*}-q}{p^{*}}}S_{\mu ,0}^{-\frac{q}{p}} \Vert u \Vert ^{q} \\ &\geq\frac{1}{N}S_{\mu,0}^{\frac{N}{p}}-D\lambda^{\frac{p}{p-q}}, \end{aligned}$$

where *D* is defined in (2.18). Hence, we conclude that $\Lambda-D\lambda^{\frac{p}{p-q}}\leq\kappa _{\lambda}^{-}<\Lambda-D\lambda^{\frac{p}{p-q}}$, which is a contradiction. It follows that $\nu_{j}=0$ for $j\in\{0\}\cup\mathcal{J}$, which means that $\int_{\Omega} \vert u_{n} \vert ^{p^{*}}\,dx\rightarrow\int_{\Omega } \vert u \vert ^{p^{*}}\,dx$ as $n\rightarrow\infty$. The proof is completed. □

In the following, we need some estimates for the extremal function $V_{\epsilon}$ defined in Lemma 2.7. Given $R>0$, let $\varphi(x)\in W_{0}^{1,p}(\Omega)$, $0\leq\varphi(x)\leq1$, $\varphi(x)=1$ for $\vert x \vert \leq R$, $\varphi(x)=0$ for $\vert x \vert \geq2R$. Set $v_{\epsilon }(x)=\varphi(x)V_{\epsilon}(x)$. For $1< p< N$ and $1< q< p^{*}$, we have the following estimates (see [4, 6]):

2.21 $$\begin{aligned} & \Vert v_{\epsilon} \Vert ^{p}=(S_{\mu,0})^{\frac{N}{p}}+O \bigl(\epsilon^{b(\mu)p+p-N}\bigr), \end{aligned}$$

2.22 $$\begin{aligned} &\int_{\Omega} \vert v_{\epsilon} \vert ^{p^{*}}\,dx=(S_{\mu,0})^{\frac {N}{p}}+O\bigl(\epsilon^{b(\mu)p^{*}-N} \bigr), \end{aligned}$$

then

2.23 $$ \int_{\Omega} \vert v_{\epsilon} \vert ^{q}\,dx=\textstyle\begin{cases} C\epsilon^{N+q(1-\frac{N}{p})} &\frac{N}{b(\mu)}< q< p, \\ C\epsilon ^{N+q(1-\frac{N}{p})} \vert \ln\epsilon \vert & q=\frac{N}{b(\mu)}, \\ C\epsilon ^{q(b(\mu)+1-\frac{N}{p})} &1< q< \frac{N}{b(\mu)}, \end{cases} $$

where $b(\mu)$ is the zero of the function

$$f(\xi)=(p-1)\xi^{p}-(N-p)\xi^{p-1}+\mu,\quad \xi\geq0, 0\leq\mu< \overline{\mu}, $$

satisfying $0<\frac{N-p}{p}<b(\mu)<\frac{N-p}{p-1}$.

### Lemma 2.9

*There exists* $\lambda_{0}>0$ *such that*

$$\sup_{s\geq0}I_{\lambda}(sv_{\epsilon})< \Lambda-D \lambda^{\frac {p}{p-q}},\quad \textit{for }\lambda\in(0,\lambda_{0}), $$

*where* Λ *and* *D* *are defined in Lemma* 2.8.

### Proof

For two positive constants $s_{0}$ and $s_{1}$ (independent of *ϵ*, *λ*), we show that there exists $s_{\epsilon}>0$ with $0< s_{0}\leq s_{\epsilon}\leq s_{1}<\infty$ such that $\sup_{s\geq0}I_{\lambda}(sv_{\epsilon})=I_{\lambda }(s_{\epsilon}v_{\epsilon})$. In fact, since $\lim_{s\rightarrow+\infty}I_{\lambda}(sv_{\epsilon })=-\infty$, we can deduce that

2.24 $$ s_{\epsilon}^{p-1} \Vert v_{\epsilon} \Vert ^{p}-\beta s_{\epsilon}^{p-1} \int _{\Omega} \vert v_{\epsilon} \vert ^{p} \vert x \vert ^{\alpha-p}\,dx- s_{\epsilon}^{p^{*}-1} \int_{\Omega} \vert v_{\epsilon} \vert ^{p^{*}}\,dx- \lambda s_{\epsilon}^{q-1} \int_{\Omega} \vert v_{\epsilon} \vert ^{q}\,dx=0 $$

and

2.25 $$\begin{aligned} &(p-1)s_{\epsilon}^{p-2} \Vert v_{\epsilon} \Vert ^{p}-(p-1)\beta s_{\epsilon}^{p-2} \int_{\Omega} \vert v_{\epsilon } \vert ^{p} \vert x \vert ^{\alpha-p}\,dx \\ &\quad{}- \bigl(p^{*}-1\bigr)s_{\epsilon}^{p^{*}-2} \int_{\Omega} \vert v_{\epsilon} \vert ^{p^{*}}\,dx -(q-1)\lambda s_{\epsilon}^{q-2} \int_{\Omega} \vert v_{\epsilon} \vert ^{q}\,dx< 0. \end{aligned}$$

Equations (2.24) and (2.25) imply that

$$\begin{aligned} &(p-1)s_{\epsilon}^{p-2} \Vert v_{\epsilon} \Vert ^{p}-(p-1)\beta s_{\epsilon}^{p-2} \int_{\Omega} \vert v_{\epsilon } \vert ^{p} \vert x \vert ^{\alpha-p}\,dx- \bigl(p^{*}-1\bigr)s_{\epsilon}^{p^{*}-2} \int_{\Omega} \vert u_{\epsilon} \vert ^{p^{*}}\,dx \\ &\quad < (q-1)s_{\epsilon}^{p-2} \Vert v_{\epsilon} \Vert ^{p}- (q-1)\beta s_{\epsilon}^{p-2} \int_{\Omega} \vert v_{\epsilon} \vert ^{p} \vert x \vert ^{\alpha-p}\,dx -(q-1)s_{\epsilon}^{p^{*}-2} \int_{\Omega} \vert v_{\epsilon} \vert ^{p^{*}}\,dx. \end{aligned}$$

That is,

2.26 $$ (p-q)s_{\epsilon}^{p-2} \Vert v_{\epsilon} \Vert ^{p}-(p-q)\beta s_{\epsilon }^{p-2} \int_{\Omega} \vert v_{\epsilon} \vert ^{p} \vert x \vert ^{\alpha-p}\,dx< \bigl(p^{*}-q\bigr)s_{\epsilon}^{p^{*}-2} \int_{\Omega} \vert v_{\epsilon} \vert ^{p^{*}}\,dx. $$

Hence, we can obtain from (2.26) that $s_{\epsilon}$ is bounded below. Moreover, it is clear to see from (2.24) that $s_{\epsilon}$ is bounded above for all $\epsilon>0$ small enough. Therefore, our claim holds.

Set

$$h(s_{\epsilon}v_{\epsilon})=\frac{s_{\epsilon}^{p}}{p} \Vert v_{\epsilon} \Vert ^{p}- \frac{s_{\epsilon}^{p^{*}}}{p^{*}} \int_{\Omega} \vert v_{\epsilon} \vert ^{p^{*}}\,dx. $$

In the following, we prove that

2.27 $$ h(s_{\epsilon}v_{\epsilon})\leq\Lambda+O\bigl( \epsilon^{p(b(\mu)-\frac{N}{p}+1)}\bigr). $$

Let

$$\widetilde{h}(s)=\frac{s^{p}}{p} \Vert v_{\epsilon} \Vert ^{p}- \frac{s^{p^{*}}}{p^{*}} \int_{\Omega} \vert v_{\epsilon} \vert ^{p^{*}}\,dx. $$

Direct computations give us that $\lim_{s\rightarrow\infty}\widetilde {h}(s)=-\infty$ and $\widetilde{h}(0)=0$. Thus $\sup_{s\geq0}\widetilde{h}(s)$ is obtained at some $S_{\epsilon }>0$, and

$$S_{\epsilon}=\biggl(\frac{ \Vert v_{\epsilon} \Vert ^{p}}{ \int_{\Omega} \vert v_{\epsilon} \vert ^{p^{*}}\,dx}\biggr)^{\frac{1}{p^{*}-p}}. $$

Since $\widetilde{h}^{\prime}(s) \vert _{S_{\epsilon}}=0 $, that is,

$$S_{\epsilon}^{p-1} \Vert v_{\epsilon} \Vert ^{p} -S_{\epsilon}^{p^{*}-1} \int_{\Omega} \vert v_{\epsilon} \vert ^{p^{*}}\,dx=0. $$

It is easy to check that $h(s)$ is increasing in $[0,S_{\epsilon})$, according to (2.21) and (2.22), we have

2.28 $$\begin{aligned} h(s_{\epsilon}v_{\epsilon})&\leq\widetilde {h}(S_{\epsilon}) \\ & =\biggl(\frac{1}{p}-\frac{1}{p^{*}}\biggr)\frac{( \Vert v_{\epsilon} \Vert ^{p})^{\frac {p^{*}}{p^{*}-p}}}{ (\int_{\Omega} \vert u_{\epsilon} \vert ^{p^{*}}\,dx)^{\frac{p}{p^{*}-p}}} \\ & =\biggl(\frac{1}{p}-\frac{1}{p^{*}}\biggr)\frac{((S_{\mu,0})^{\frac{N}{p}} +O(\epsilon^{b(\mu)p+p-N}))^{\frac{p^{*}}{p^{*}-p}}}{((S_{\mu,0})^{\frac{N}{p}} +O(\epsilon^{b(\mu)p^{*}-N}))^{\frac{p}{p^{*}-p}}} \\ &\leq\biggl(\frac{1}{p}-\frac{1}{p^{*}}\biggr) \frac{(S_{\mu,0})^{\frac{N}{p}\frac{p^{*}}{p^{*}-p}}}{(S_{\mu,0}) ^{\frac{N}{p}\frac{p}{p^{*}-p}}}+O \bigl(\epsilon^{b(\mu)p+p-N}\bigr) \\ &=\biggl(\frac{1}{p}-\frac{1}{p^{*}}\biggr) (S_{\mu,0})^{\frac{N}{p}}+O \bigl(\epsilon^{p(b(\mu)-\frac{N}{p}+1)}\bigr) \\ &=\Lambda+O\bigl(\epsilon^{p(b(\mu)-\frac{N}{p}+1)}\bigr). \end{aligned}$$

Therefore, by (2.27), we have

2.29 $$\begin{aligned} I_{\lambda}(s_{\epsilon}v_{\epsilon})&=h(s_{\epsilon} v_{\epsilon})-\frac{\beta s_{\epsilon}^{p}}{p} \int_{\Omega} \vert v_{\epsilon} \vert ^{p} \vert x \vert ^{\alpha-p}\,dx-\frac{\lambda s_{\epsilon}^{q}}{q} \int_{\Omega} \vert v_{\epsilon} \vert ^{q}\,dx \\ &\leq\Lambda+C\epsilon^{p(b(\mu)-\frac{N}{p}+1)}-\frac{\beta}{p}s_{0}^{p} \int_{\Omega} \vert v_{\epsilon} \vert ^{p} \vert x \vert ^{\alpha-p}\,dx -\frac{\lambda s_{0}^{q}}{q} \int_{\Omega} \vert v_{\epsilon} \vert ^{q}\,dx. \end{aligned}$$

Now, we consider the following cases:

(i) $\frac{N}{b(\mu)}< q< p$. Choose $\epsilon=\lambda^{\frac{1}{(p-q)(b(\mu)-\frac{N}{p}+1)}}$, for $\lambda<\lambda_{1}:=(\frac{C_{1}+D}{C_{2}})^{\frac{(p-q)(b(\mu)-\frac {N}{p}+1)}{N-qb(\mu)}}$, we have

$$\begin{aligned} C_{1} \epsilon^{p(b(\mu)-\frac{N}{p}+1)}-\lambda C_{2} \epsilon^{N+q(1-\frac{N}{p})} & =C_{1} \lambda^{\frac{p}{p-q}}-\lambda C_{2} \lambda^{\frac{N+q(1-\frac {N}{p})}{(p-q)(b(\mu)-\frac{N}{p}+1)}} \\ & =C_{1} \lambda^{\frac{p}{p-q}}-C_{2} \lambda^{\frac{N+q(1-\frac {N}{p})}{(p-q)(b(\mu)-\frac{N}{p}+1)}+1} \\ & =\lambda^{\frac{p}{p-q}}\bigl(C_{1}-C_{2} \lambda^{\frac{N-qb(\mu)}{(p-q)(b(\mu )-\frac{N}{p}+1)}}\bigr) \\ & < -D \lambda^{\frac{p}{p-q}}. \end{aligned}$$

(ii) $q=\frac{N}{b(\mu)}$. We still choose $\epsilon=\lambda^{\frac{1}{(p-q)(b(\mu)-\frac {N}{p}+1)}}$, for $\lambda<\lambda_{2}:= e^{-(\frac{C_{1} +D}{C_{3}})}$, we have

$$\begin{aligned} C_{1} \epsilon^{p(b(\mu)-\frac{N}{p}+1)}-\lambda C_{2} \epsilon^{N+q(1-\frac{N}{p})} \vert \ln\epsilon \vert & =C_{1} \lambda^{\frac{p}{p-q}}-\lambda C_{3} \lambda^{\frac{N+q(1-\frac {N}{p})}{(p-q)(b(\mu)-\frac{N}{p}+1)}} \vert \ln\lambda \vert \\ & =C_{1} \lambda^{\frac{p}{p-q}}-C_{3} \lambda^{\frac{N+q(1-\frac {N}{p})}{(p-q)(b(\mu)-\frac{N}{p}+1)}+1} \vert \ln\lambda \vert \\ & < \lambda^{\frac{p}{p-q}}\bigl(C_{1} -C_{3} \vert \ln \lambda \vert \bigr) \\ & < -D\lambda^{\frac{p}{p-q}}, \end{aligned}$$

where $C_{3} =\frac{C_{2}}{(p-q)(b(\mu)-\frac{N}{p}+1)}$.

(iii) $1< q<\frac{N}{b(\mu)}$. Put $\epsilon^{p(b(\mu)-\frac {N}{p}+1)}\leq\lambda^{\frac{p}{p-q}}$, for $\lambda<\lambda_{3}:=(\frac{C_{2} -D}{C_{1}})^{\frac{p-q}{pq-p}}$ with $C_{2} >D$, we have

$$\begin{aligned} C_{1} \epsilon^{p(b(\mu)-\frac{N}{p}+1)}-\lambda C_{2} \epsilon^{q(b(\mu)+1-\frac{N}{p})} & :=C_{1} \lambda^{\frac{pq}{p-q}}-\lambda C_{2} \lambda^{\frac{q}{p-q}} \\ & =\lambda^{\frac{p}{p-q}}\bigl(C_{1} \lambda^{\frac{pq-p}{p-q}}-C_{2} \bigr) \\ & < -D\lambda^{\frac{p}{p-q}}. \end{aligned}$$

Consequently, for $\lambda<\lambda_{0}:=\min\{\lambda_{1}, \lambda_{2}, \lambda_{3}\}$, we deduce that

$$I_{\lambda}(s_{\epsilon}v_{\epsilon})< \Lambda-D \lambda^{\frac{p}{p-q}}. $$

 □

## Proof of main result

We can find a constant $\delta>0$ such that $\Lambda-D\lambda^{\frac {p}{p-q}}>0$ for $\lambda<\delta$. Let $\lambda_{*}=\min\{T_{1}, \delta, \lambda_{0}\}$. For $\lambda\in(0,\lambda_{*})$, Lemmas 2.1-2.4, 2.6 and 2.8 hold.

Let $\{u_{n}\}\subset\mathcal{N}_{\lambda}$ be a minimizing sequence of $I_{\lambda}$. It is easy to see that $\{u_{n}\}$ is bounded in $W_{0}^{1,p}(\Omega)$ and there exist a subsequence of $\{u_{n}\}$ (still denoted by $\{u_{n}\}$) and $u_{\lambda}\in W_{0}^{1,p}(\Omega)$ such that

3.1 $$ \textstyle\begin{cases} u_{n}\rightharpoonup u_{\lambda} \quad\mbox{weakly in }W_{0}^{1,p}(\Omega), \\ u_{n}\rightarrow u_{\lambda} \quad\mbox{strongly in }L^{s}(\Omega)\ (1\leq s< p^{*}), \\ u_{n}(x)\rightarrow u_{\lambda}(x) \quad\mbox{a.e. in }\Omega, \end{cases} $$

as $n\rightarrow\infty$.

Firstly, by Lemma 2.4, we can know that $f_{n}^{\prime}(0)$ is bounded with respect to $n\in\mathbb{N}$. Letting $n\rightarrow\infty$ in (2.11), we deduce that

3.2 $$\begin{aligned} &\int_{\Omega} \vert \nabla u_{*} \vert ^{p-2}\nabla u_{*}\cdot\nabla\phi-\mu \int _{\Omega}\frac{ \vert u_{*} \vert ^{p-2}u_{*}}{ \vert x \vert ^{p}}\phi \\ &\quad= \int_{\Omega } \vert u_{*} \vert ^{p^{*}-1}\phi +\lambda \int_{\Omega} \vert u_{*} \vert ^{q-1} \phi+\beta \int_{\Omega } \vert u_{*} \vert ^{p-1} \vert x \vert ^{\alpha-p} \phi \end{aligned}$$

for all $\phi\in W_{0}^{1,p}(\Omega)$. Equation (3.2) implies that $u_{\lambda}$ is a solution of (1.1). We claim that $u_{\lambda}\not\equiv0$. If not, $u_{\lambda}=0$, since $u_{n}\in\mathcal{N}_{\lambda}$, we have

$$\Vert u_{n} \Vert ^{p} - \int_{\Omega} \vert u_{n} \vert ^{p^{*}}- \beta \int_{\Omega} \vert u_{n} \vert ^{p} \vert x \vert ^{\alpha -p}-\lambda \int_{\Omega} \vert u_{n} \vert ^{q} =0. $$

Note that

$$\lim_{n\rightarrow\infty} \int_{\Omega} \vert u_{n} \vert ^{p} \vert x \vert ^{\alpha-p}\,dx=0,\qquad \lim_{n\rightarrow\infty} \int_{\Omega} \vert u_{n} \vert ^{q} \,dx=0. $$

Put $\lim_{n\rightarrow\infty} \Vert u_{n} \Vert =m$, we conclude that $m\geq S_{\mu,0}^{\frac{p^{*}}{p(p^{*} -p)}}$. By Lemma 2.8, we obtain

$$\begin{aligned} \kappa_{\lambda}&=\lim_{n\rightarrow\infty}I_{\lambda}(u_{n}) \\ &= \lim_{n\rightarrow\infty}\biggl[\frac{1}{p} \Vert u_{n} \Vert ^{p} -\frac{\beta}{p} \int _{\Omega} \vert u_{n} \vert ^{p} \vert x \vert ^{\alpha-p} -\frac{1}{p^{*}} \int_{\Omega} \vert u_{n} \vert ^{p^{*}}\,dx- \frac{\lambda}{q} \int_{\Omega} \vert u_{n} \vert ^{q} \,dx \biggr] \\ &\geq \lim_{n\rightarrow\infty}\biggl(\frac{1}{p}-\frac{1}{p^{*}} \biggr) \Vert u_{n} \Vert ^{p} \\ &\geq\frac{p^{*} -p}{p p^{*}}S_{\mu,0}^{\frac{p^{*}}{p^{*} -p}} \\ & =\frac{1}{N}S_{\mu,0}^{\frac{N}{p}}, \end{aligned}$$

which contradicts with $\kappa_{\lambda}<\Lambda-D\lambda^{\frac{p}{p-q}}$ (from Lemma 2.9).

Secondly, we prove that $u_{\lambda}\in\mathcal{N}_{\lambda}^{+}$. Suppose that this is not true, $i.e$., $u_{\lambda}\in\mathcal {N}_{\lambda}^{-}$. From Lemma 2.1, we can find positive numbers $s^{+}$ and $s^{-}$ with $s^{+}< s_{\max}< s^{-}=1$ such that $s^{+}u_{\lambda}\in \mathcal{N}_{\lambda}^{+}$, $s^{-}u_{\lambda}\in\mathcal{N}_{\lambda }^{-}$ and

$$\kappa_{\lambda}< I_{\lambda}\bigl(s^{+}u_{\lambda} \bigr)< I_{\lambda }\bigl(s^{-}u_{\lambda} \bigr)=I_{\lambda}(u_{\lambda})=\kappa_{\lambda}, $$

which is a contradiction. Hence $u_{\lambda}\in\mathcal{N}_{\lambda}^{+}$. Furthermore, combining with Lemma 2.3, we can obtain

$$I_{\lambda}(u_{\lambda})=\kappa_{\lambda}^{+}= \kappa_{\lambda}< 0. $$

Therefore, we see that $u_{\lambda}$ is a non-negative ground state solution of problem (1.1).

In the following, we prove that problem (1.1) has a second solution $v_{\lambda}$ with $v_{\lambda}\in\mathcal{N}_{\lambda}^{-}$. Since $I_{\lambda}$ is coercive on $\mathcal{N}_{\lambda}^{-}$, according to the Ekeland variational principle and Lemma 2.9, there exists a minimizing sequence $\{v_{n}\}\subset\mathcal {N}_{\lambda}^{-}$ of $I_{\lambda}$ such that

(i) $I_{\lambda}(v_{n})<\kappa_{\lambda}^{-}+\frac{1}{n}$;
(ii) $I_{\lambda}(u)\geq I_{\lambda}(v_{n})-\frac{1}{n} \Vert u-v_{n} \Vert $ for all $u\in\mathcal{N}_{\lambda}^{-}$.

Note that $\{v_{n}\}$ is bounded in $W_{0}^{1,p}(\Omega)$, there exist a subsequence (still denoted by $\{v_{n}\}$) and $v_{\lambda}\in W_{0}^{1,p}(\Omega)$ such that

3.3 $$ \textstyle\begin{cases} v_{n}\rightharpoonup v_{\lambda} \quad\mbox{weakly in }W_{0}^{1,p}(\Omega), \\ v_{n}\rightarrow v_{\lambda}\quad \mbox{strongly in }L^{s}(\Omega)\ (1\leq s< p^{*}), \\ v_{n}(x)\rightarrow v_{\lambda}(x) \quad\mbox{a.e. in }\Omega, \end{cases} $$

as $n\rightarrow\infty$.

Similar to the above discussion, we can deduce that $v_{n}\rightarrow v_{\lambda}$ in $W_{0}^{1,p}(\Omega)$ and $v_{\lambda}$ is a non-negative solution of (1.1). Thirdly, we show that $v_{\lambda}\neq0$ in Ω. According to $v_{n}\in\mathcal{N}_{\lambda}^{-}$, we obtain

$$\begin{aligned} (p-q) \Vert v_{n} \Vert ^{p}&=\bigl(p^{*}-q \bigr) \int_{\Omega} \vert v_{n} \vert ^{p^{*}}\,dx+(p-q) \beta \int_{\Omega} \vert v_{n} \vert ^{p} \vert x \vert ^{\alpha-p}\,dx \\ & < \bigl(p^{*}-q\bigr)S_{\mu,0}^{-\frac{p^{*}}{p}} \Vert v_{n} \Vert ^{p^{*}} +(p-q)\frac{\beta}{\beta_{1}} \Vert v_{n} \Vert ^{p}, \end{aligned}$$

hence

3.4 $$ \Vert v_{n} \Vert >\biggl[\frac{(p-q)(1-\frac{\beta}{\beta_{1}})S_{\mu,0}^{\frac{p^{*}}{p}}}{ p^{*}-q} \biggr]^{\frac{1}{p^{*}-p}}, \quad\forall v_{n}\in\mathcal{N}_{\lambda}^{-}, $$

together with $v_{n}\rightarrow v_{\lambda}$ in $W_{0}^{1,p}(\Omega)$ means that $v_{\lambda}\not\equiv0$.

Lastly, we show that $v_{\lambda}\in\mathcal{N}_{\lambda}^{-}$. We only need to prove that $\mathcal{N}_{\lambda}^{-}$ is closed. In fact, for $\{v_{n}\}\subset\mathcal{N}_{\lambda}^{-}$, it follows from Lemmas 2.8 and 2.9 that

$$\lim_{n\rightarrow\infty} \int_{\Omega} \vert v_{n} \vert ^{p^{*}}\,dx= \int_{\Omega} \vert v_{\lambda} \vert ^{p^{*}}\,dx. $$

In addition

$$(p-q) \Vert v_{n} \Vert ^{p}-\bigl(p^{*}-q \bigr) \int_{\Omega} \vert v_{n} \vert ^{p^{*}}\,dx-(p-q)\beta \int_{\Omega} \vert v_{n} \vert ^{p} \vert x \vert ^{\alpha-p}\,dx< 0. $$

Thus

$$(p-q) \Vert v_{\lambda} \Vert ^{p}-\bigl(p^{*}-q \bigr) \int_{\Omega} \vert v_{\lambda} \vert ^{p^{*}}\,dx-(p-q)\beta \int_{\Omega} \vert v_{\lambda} \vert ^{p} \vert x \vert ^{\alpha-p}\,dx\leq0, $$

which means that $v_{\lambda}\in\mathcal{N}_{\lambda}^{0}\cup\mathcal {N}_{\lambda}^{-}$. Combining with Lemma 2.1 and $v_{\lambda}\not\equiv0$, we see that $\mathcal{N}_{\lambda}^{-}$ is closed. Note that $\mathcal{N}_{\lambda}^{+}\cap\mathcal{N}_{\lambda }^{-}=\emptyset$, we know that $u_{\lambda}$ and $v_{\lambda}$ are different.

## Conclusions

In this paper, we study the existence and multiplicity of positive solutions for the quasi-linear elliptic problem which consists of critical Sobolev exponent and a Hardy term.

The main conclusions of this work:

1. Adding a linear perturbation in the nonlinear term of elliptic equation.
2. The main challenge of this study is the lack of compactness of the embedding $W_{0}^{1,p}\hookrightarrow L^{p^{*}}$. We overcome it by the concentration compactness principle.
3. We apply the Ekeland variational principle to obtain a minimizing sequence with good properties.

## Discussion

In the future, a natural question is whether the multiplicity of positive solutions for (1.1) can be established with negative exponent $\frac{1}{u^{\gamma}}\ (0<\gamma<1)$.
